# A Revised Comorbidity Model for Administrative Databases Using Clinical Classifications Software Refined Variables

**DOI:** 10.7759/cureus.20407

**Published:** 2021-12-14

**Authors:** Hafeez Shaka, Ehizogie Edigin

**Affiliations:** 1 Internal Medicine, John H. Stroger, Jr. Hospital of Cook County, Chicago, USA; 2 Rheumatology, Loma Linda University Medical Center, Loma Linda, USA

**Keywords:** ccsr, comorbidity models, mortality index, hospital outcomes, database study

## Abstract

Background and objective

Database research has shaped policies, identified trends, and informed healthcare guidelines for numerous disease conditions. However, despite their abundant uses and vast potential, administrative databases have several limitations. Adjusting outcomes for comorbidities is often needed during database analysis as a means of overcoming non-randomization. We sought to obtain a model for comorbidity adjustment based on Clinical Classifications Software Refined (CCSR) variables and compare this with current models. Our aim was to provide a simplified, adaptable, and accurate measure for comorbidities in the Agency for Healthcare Research and Quality (AHRQ) databases, in order to strengthen the validity of outcomes.

Methods

The Nationwide Inpatient Sample (NIS) database for 2018 was the data source. We obtained the mortality rate among all included hospitalizations in the dataset. A model based on CCSR categories was mapped from disease groups in Sundararajan's adaptation of the modified Deyo’s Charlson Comorbidity Index (CCI). We employed logistic regression analysis to obtain the final model using CCSR variables as binary variables. We tested the final model on the 10 most common reasons for hospitalizations.

Results

The model had a higher area under the curve (AUC) compared to the three modalities of the CCI studied in all the categories. Also, the model had a higher AUC compared to the Elixhauser model in 8/10 categories. However, the model did not have a higher AUC compared to a model made from stepwise backward regression analysis of the original 21-variable model.

Conclusion

We developed a 15-CCSR-variable model that showed good discrimination for inpatient mortality compared to prior models.

## Introduction

Database research has been instrumental in shaping policies, identifying trends, and informing healthcare guidelines for numerous disease conditions [1-6]. A majority of databases, including the Agency for Healthcare Research and Quality (AHRQ) databases, are coded using International Classification of Diseases (ICD) codes. Despite their abundant uses and vast potential, administrative databases have several limitations related to coding, missing data, inadequate classification, among others. The difficulty in clinical translation of findings from retrospective and non-randomized databases is a unique challenge facing these databases [7-10].

Adjusting outcomes for comorbidities is often needed during database analysis as a means of overcoming non-randomization. Various approaches have been employed in the literature to this end, including the use of index scoring or individual comorbidities [11,12]. The Charlson Comorbidity Index (CCI) and the Elixhauser Comorbidity Index (ECI) are the most common indices used as comorbidity measures in administrative databases [13,14]. These have undergone various modifications and adaptations to suit the changing ICD iterations and specific medical conditions [15-17]. Researchers have had to develop individual comorbidity adjustment methods, which makes reproducibility very challenging. This is often due to nonuniformity in diagnostic codes attributed to comorbidities. The extent to which the CCI and ECI models adjust for individual conditions is the subject of ongoing debate, as there has been substantial improvement in healthcare since they were initially modeled. For example, the CCI attributes a six-fold increase in mortality for a patient with HIV infection compared to heart failure. The current version of the ECI contains 39 variables and requires specialized software to analyze. A large number of variables means analyses are subject to overfitting.

The latest AHRQ databases incorporate the Clinical Classifications Software Refined (CCSR) categories into datasets. This aids in the standardized mapping of diseases into clinically relevant categories. In this study, we sought to obtain a model for comorbidity adjustment based on the incorporated CCSR variables and compare this with current models. Our objective was to provide a simplified, adaptable, and accurate measure for comorbidities in AHRQ databases, which would strengthen the validity of outcomes.

## Materials and methods

Data source

The Nationwide Inpatient Sample (NIS) database for 2018 was the data source. The NIS is developed by the Healthcare Cost and Utilization Project (HCUP), a federal-state-industry partnership sponsored by the AHRQ. It is a registry of hospital inpatient stays derived from billing data submitted by hospitals to statewide data organizations across the US, covering more than 97% of the US population [18]. The 2018 database was coded using the ICD, Tenth Revision, Clinical Modification/Procedure Coding System (ICD-10-CM/PCS). In the NIS, diagnoses are divided into principal diagnosis and secondary diagnosis. A principal diagnosis was the main ICD-10 code for hospitalization. Secondary diagnoses were any ICD-10 code other than the principal diagnosis. Since 2018, HCUP databases have included the Diagnosis and Procedure Groups (DPG) file, and this includes data elements derived from the CCSR for ICD-10-CM [18]. The CCSR for ICD-10-CM diagnoses aggregates more than 70,000 ICD-10-CM diagnosis codes into over 530 clinically meaningful categories. The CCSR for ICD-10-CM diagnosis provides a means by which to identify specific clinical conditions using ICD-10-CM diagnosis codes [19]. The 2018 database contains over seven million unweighted hospitalization stays. We excluded hospitalizations involving patients aged less than 18 years and those with missing values for age, sex, and disposition.

Outcome measures

We obtained the mortality rate of all included hospitalizations in the dataset. A model based on CCSR categories present in the DPG file was mapped from disease groups in the Sundararajan’s adaptation of the modified Deyo’s CCI [15]. We included smoking history, obesity, malnutrition, and anemia as variables that have impacted mortality in prior HCUP studies [20-22]. Mortality is a common outcome of administrative database analysis, which has demonstrated high reliability in coding [23].

The Clinical Classifications Software Refined variables

Table 1 shows the 21 CCSR variables included in the initial model. The variables were coded as binary parameters among the hospitalizations. Each data element DXCCSR_AAAnnn identifies whether the CCSR category was triggered by a diagnosis code on the record. The value of AAA indicates the body system. The value of nnn indicates the specific category within the body system. For each CCSR variable included, a recorded value of 3 means the CCSR was triggered by only secondary diagnosis code(s) on the input record [24]. This was used to determine the comorbidity burden of hospitalizations. The exact ICD-10 mapping of these categories is also provided by the HCUP to ensure uniformity during data analysis.

**Table 1:** CCSR variables from the Charlson Comorbidity Index modification *Excluded from the final model CCSR: Clinical Classifications Software Refined; COPD: chronic obstructive pulmonary disease

CCSR variables	CCSR codes
Acute myocardial infarction	DXCCSR_CIR009
Congestive heart failure	DXCCSR_CIR019
Peripheral vascular disease	DXCCSR_CIR026
Cerebral infarction	DXCCSR_CIR020
Dementia/neurocognitive disorder	DXCCSR_NVS011
Pulmonary disease (asthma, COPD, pneumoconiosis)	DXCCSR_RSP008, DXCCSR_RSP009, DXCCSR_RSP013
Connective tissue disorder/rheumatologic*	DXCCSR_MUS003, DXCCSR_MUS008, DXCCSR_MUS024
Peptic ulcer disease*	DXCCSR_DIG005
Liver disease	DXCCSR_DIG019, DXCCSR_DIG023
Diabetes without complications*	DXCCSR_END002
Diabetes complications*	DXCCSR_END003
Paraplegia/paralysis	DXCCSR_NVS008
Renal disease	DXCCSR_GEN003
Cancer	DXCCSR_NEO001 ‐ DXCCSR_NEO069, DXCCSR_NEO071
Metastatic cancer	DXCCSR_NEO070
Severe liver disease/hepatic failure	DXCCSR_DIG018
Human immunodeficiency virus*	DXCCSR_INF006
Obesity	DXCCSR_END009
Malnutrition	DXCCSR_END008
Smoking history	DXCCSR_MBD024
Anemia*	DXCCSR_BLD001 ‐ DXCCSR_BLD005

Statistical analysis

We employed logistic regression analysis to obtain the final model using CCSR variables as binary variables. Since the dataset has over six million hospitalizations, we bootstrapped 100 replications of a 5% sample for mortality, as employed by Moore et al. [25], while employing stepwise backward regression. This was done to avoid overpowering and avoid variables attaining statistical significance while only marginally changing the outcome. We subsequently included variables with p-values <0.01 in the final model. We tested for collinearity among the included variables using the variance-covariance matrix estimation to obtain covariates. We tested the predictive power of the model using the c-statistic, expressed as area under the curve (AUC).

Model validation

We tested the final model on the 10 most common reasons for hospitalizations as analyzed by Moore et al. [25]. Diagnoses of hospitalizations were mapped using CCSR codes for any hospitalization with a principal diagnosis of the conditions. For each CCSR-mapped principal diagnosis, we compared the c-statistics of the final 15-factor model, against individual stepwise backward regression using the initial 21 CCSR variables, individual CCI weights, total CCI, grouped CCI, and the Elixhauser model for mortality. The CCI was grouped into 0, 1, 2, and ≥3. The c-statistic for the final model and a model with grouped age was compared to that of the Elixhauser model. The backward stepwise selection involved removing terms with p≥0.2 and adding those with p<0.1. All analyses were performed using the unweighted dataset.

Ethical considerations

The NIS database lacks patient-level identifiers. Hence, this study did not require any institutional review board approval.

Data availability statement

The NIS is a large, publicly available, all-payer inpatient care database in the US, containing data on more than seven million hospital stays yearly. Its large sample size makes it ideal for developing national and regional estimates and enables analyses of rare conditions, uncommon treatments, and special populations.

## Results

Final CCSR model

Table 2 shows the 15-CCSR-based variable model obtained. Following bootstrapped analysis and backward stepwise regression, we excluded connective tissue/rheumatologic disorders, peptic ulcer disease, anemia, diabetes without complications, diabetes with complications, and human immunodeficiency as predictors of inpatient mortality. Pulmonary disease was the most prevalent condition among hospitalized patients. Liver failure had the highest impact on mortality [adjusted odds ratio (aOR): 11.75 (11.49-12.02)]. However, smoking history and obesity were associated with lower odds of inpatient mortality. The c-statistic for the final model had an AUC of 0.784.

**Table 2:** Final CCSR variable model showing proportion and effect on mortality aOR: adjusted odds ratio for mortality; CCSR: Clinical Classifications Software Refined; COPD: chronic obstructive pulmonary disease

CCSR variables	Proportion, %, n=6048698	aOR (95% confidence interval)
Acute myocardial infarction	1.66	3.94 (3.86–4.03)
Congestive heart failure	13.81	2.18 (2.15–2.20)
Peripheral vascular disease	4.29	1.51 (1.48–1.54)
Cerebral infarction	0.74	3.90 (3.78–4.03)
Dementia/neurocognitive disorder	7	1.86 (1.83–1.89)
Pulmonary disease (asthma, COPD, pneumoconiosis)	20.42	1.28 (1.27–1.30)
Liver disease	4.35	1.17 (1.14–1.20)
Paraplegia/paralysis	2.2	2.10 (2.04–2.15)
Renal disease	15.3	1.43 (1.42–1.46)
Cancer	6.07	1.88 (1.85–1.91)
Metastatic cancer	2.7	2.85 (2.78–2.91)
Severe liver disease/hepatic failure	1.03	11.75 (11.49–12.02)
Obesity	16.61	0.68 (0.67–0.69)
Malnutrition	5.89	2.30 (2.26–2.33)
Smoking history	16.7	0.62 (0.60–0.63)

Model validation

Table 3 represents a comparison of the AUC obtained from the 15-variable model (CCSR bootstrapped model) with other models across 10 diagnostic categories. The model had a higher AUC compared to the three modalities of the CCI studied in all the categories. Additionally, the model had a higher AUC compared to the Elixhauser model in 8/10 categories. However, the model did not have a higher AUC compared to a model made from stepwise backward regression analysis of the original 21-variable model. This was most significant in the models for congestive heart failure (0.560 vs. 0.693).

**Table 3:** Comparison of CCSR model with other models in mortality outcomes among top 10 diagnostic categories of hospitalizations CCSR: Clinical Classifications Software Refined; CCI: Charlson Comorbidity Index; STD: sexually transmitted diseases; SW: stepwise backward; TB: tuberculosis

CCSR categories	Mortality, %	CCSR bootstrapped model	CCSR model with SW	CCI weights	CCI total	CCI grouped	Elixhauser model
Septicemia (except in labor)	8.99	0.738	0.738	0.688	0.639	0.623	0.66
Respiratory failure; insufficiency; arrest	9.36	0.74	0.74	0.7	0.575	0.579	0.623
Acute cerebrovascular disease	3.94	0.677	0.684	0.645	0.593	0.551	0.598
Pneumonia (except caused by TB or STD)	2.46	0.745	0.748	0.701	0.647	0.625	0.72
Acute myocardial infarction	4.58	0.733	0.735	0.661	0.626	0.605	0.709
Congestive heart failure	2.6	0.56	0.693	0.613	0.548	0.531	0.684
Aspiration pneumonitis	7.12	0.651	0.653	0.619	0.57	0.556	0.61
Acute and unspecified renal failure	2.3	0.759	0.76	0.721	0.636	0.598	0.706
Secondary malignancies	5.51	0.679	0.685	0.599	0.578	0.5	0.657
Traumatic brain injury	8.76	0.613	0.638	0.571	0.509	0.535	0.64

## Discussion

Our study demonstrated that the 15-CCSR-variable model for comorbidity adjustment is superior to the current CCI-based models and outperforms the ECI in a majority of the conditions analyzed while being simpler to implement. The ease of reproducibility is another advantage of our model. However, we noted significant variability in the model validation between the individual conditions ranging from a c-statistic of 0.560 for congestive heart failure to 0.759 for acute and unspecified renal failure. This translates into fair to very good discrimination as predictive models.

We also discovered that employing a stepwise backward regression to the original 21-CCSR-variable model for the individual conditions was superior to the 15-CCSR-variable model. This allows for individual weighting of comorbidities for a particular condition. Although research by Austin et al. [26] suggests that indexing works, disease-specific models continue to demonstrate superior discrimination as predictive models. Our study again demonstrated this with the relatively poorer performance of the CCI total or CCI grouped, compared to CCI weights. All the CCI models were mostly less discriminant than the CCSR models. This is likely due to the outdated weighting and inclusion of variables that do not have the same impact on mortality as they once did. For instance, the advent of antiretroviral therapy has revolutionized HIV management and the incidence of AIDS. 

We noticed that the aOR for mortality for individual comorbidities varied from one condition to another. The stepwise backward regression also excluded different comorbidities while analyzing different conditions. Hence, a model that provides weights to comorbid variables would not adequately account for this variation. Consequently, the 15-CCSR variable model was retained as individual variables and not converted into a weighted index. To our knowledge, this is the first study modeling comorbidity-adjustment based on CCSR variables, which are newly included in HCUP databases.

The addition of biodemographic data such as age, sex, race, household income, primary payer, and hospital characteristics such as hospital location and size is expected to improve the CCSR-based model as observed in prior studies [14-16].

Our study has some limitations. Primarily, some CCSR variables that may impact the primary outcome may have been left out of the initial 21-variable model as the literature review to identify variables that impact inpatient mortality was not exhaustive. Another limitation is that the study retains limitations of administrative databases, such as non-randomization, under-coding, and poor classification of disease severity, which may affect mortality. The identification of comorbidities was done without the use of admission indicators to separate comorbid conditions from complications of care that develop during the hospital stay. The ECI model used for comparison was adopted from a study done using a 2011 database, which would likely have different patient characteristics. A 15-variable model could still be subject to overfitting in conditions with small population size, or with very low inpatient mortality, compared to an indexed model.

## Conclusions

Administrative databases continue to be an important part of healthcare research. The inclusion of CCSR variables in AHRQ databases provides an opportunity to develop a standardized and reproducible measure of comorbidity for various disease conditions. We developed a 15-CCSR-variable model that showed good discrimination for inpatient mortality compared to prior models. However, a disease-specific model continues to demonstrate superiority in outcomes-based research.
